# Calcitonin Gene‐Related Peptide–Expressing Sensory Neurons and Spinal Microglial Reactivity Contribute to Pain States in Collagen‐Induced Arthritis

**DOI:** 10.1002/art.39082

**Published:** 2015-05-25

**Authors:** Francisco R. Nieto, Anna K. Clark, John Grist, Victoria Chapman, Marzia Malcangio

**Affiliations:** ^1^ King's College LondonLondonUK; ^2^ University of NottinghamNottinghamUK

## Abstract

**Objective:**

To evaluate the contribution of sensory neurons in ankle joints and adjacent tissue to the development of pain in collagen‐induced arthritis (CIA), and to determine the relationship between pain and the appearance of clinical signs.

**Methods:**

Mechanical and heat hypersensitivity and hind paw swelling were assessed in Lewis rats before and until 18 days following collagen immunization. We examined the effect of intrathecal administration of a calcitonin gene‐related peptide (CGRP) antagonist (CGRP^8–37^) from day 11 to day 18 postimmunization on CIA‐induced hypersensitivity. During CIA development, CGRP and p‐ERK immunoreactivity was quantified in lumbar dorsal root ganglia in which sensory neurons innervating the ankle joint were identified by retrograde labeling with Fluoro‐Gold. Microgliosis in the lumbar dorsal horn was assessed by immunohistochemistry, and release of CGRP evoked by activity of primary afferent fibers was measured using a preparation of isolated dorsal horn with dorsal roots attached.

**Results:**

CIA was associated with mechanical hypersensitivity that was evident before hind paw swelling and that was exacerbated with the development of swelling. Heat hyperalgesia developed along with swelling. Concomitant with the development of mechanical hypersensitivity, joint innervating neurons exhibited enhanced CGRP expression and an activated phenotype (increased p‐ERK expression), and significant microgliosis became evident in the dorsal horn; these peripheral and central changes were augmented further with disease progression. CGRP release evoked by dorsal root stimulation was higher in the dorsal horn on day 18 in rats with CIA compared to control rats. Prolonged intrathecal administration of CGRP^8–37^ attenuated established mechanical hypersensitivity and reduced spinal microgliosis.

**Conclusion:**

Sensory neuron–derived CGRP sustains mechanical hypersensitivity and spinal microglial reactivity in CIA, suggesting that central mechanisms play critical roles in chronic inflammatory pain. Blockade of these central events may provide pain relief in rheumatoid arthritis patients.

Pain is the most dominant and impairing symptom associated with rheumatoid arthritis (RA). Patients may experience pain before clinical signs permit confirmation of the diagnosis of RA 1; thus, pain is present at the predisease stage as well as during the disease stage of RA. Treatment of RA pain with nonsteroidal antiinflammatory drugs (NSAIDs) results in modest efficacy and may produce side effects 2. Improved understanding of the specific mechanisms of RA‐associated pain will enable us to identify new strategies for analgesia.

Collagen‐induced arthritis (CIA) is a clinically relevant model of RA. The immunopathogenesis of CIA involves both B and T lymphocyte responses, with the production of type II collagen–specific antibodies that bind to cartilage in the joints 3. The resulting pathogenesis shares several pathologic features with RA, including synovial hyperplasia, inflammatory cell infiltration, and cartilage degradation 3. However, only a few studies (by our group and others) have investigated the mechanisms underlying pain in this model in either mice 4 or rats 5.

Although cartilage is not innervated, inflammation of the synovial membrane and bone alterations can lead to the sensitization of primary afferent fibers (nociceptors) that innervate the joints and tissue outside the joints (peripheral sensitization) and respond to noxious stimuli 6. All nociceptors contain glutamate, while the peptidergic subpopulation of nociceptors also contains substance P (SP) and calcitonin gene‐related peptide (CGRP) 7, 8 and is especially enriched in the joint 9. Increased input from such sensitized afferent fibers, whose cell bodies are located in the dorsal root ganglia (DRGs), can lead to an augmented release of glutamate, SP, and CGRP from their central terminals in the spinal cord 10, whereby increased activation of specific receptors in dorsal horn neurons results in amplification of signaling (central sensitization) 6. Along with neurons, spinal microglia are known to respond to increased neuronal activity and play modulatory roles by releasing pronociceptive mediators 11, 12, 13, 14. Central sensitization can contribute to secondary hyperalgesia in joint‐adjacent tissue (hind paw), as many spinal cord neurons receive convergent inputs from skin and deep tissues 6, 13.

Our recent work demonstrated that the early stages of CIA are associated with increased nocifensive behavior prior to the appearance of clinical signs of the disease, whereas at later stages nocifensive responses are present concomitant with significant hind paw swelling and enhanced spinal microglial response 5. The weak correlation between pain and swelling in the early stages of CIA mimics the clinical situation 1 and suggests that mechanisms other than overt inflammation contribute to pain at this stage. Thus, in this study we evaluated whether nociceptive sensory neurons innervating the joint and adjacent tissues are recruited and activated during the development of pain and inflammation in CIA and contribute to associated spinal mechanisms.

## MATERIALS AND METHODS

### Animals

Experiments were performed in 70 female adult Lewis rats weighing 180–200 gm (Charles River UK). Experimental study groups were randomized, and assessments were performed under blinded conditions. All experiments were undertaken with approval of the UK Home Office.

### CIA

As described previously 5, 4 mg/ml bovine type II collagen (MD Bioproducts) was dissolved in 0.1*M* acetic acid and emulsified with 1 mg/ml Freund's complete adjuvant (CFA; BD Biosciences). Rats were anesthetized with isoflurane (Abbott) and injected intradermally at the base of the tail with 400 μg collagen/200 μl CFA or 200 μl CFA emulsion (control rats).

### Macroscopic assessment of arthritis

Rats were scored on a scale of 0–3 per hind paw (0–6 per rat) 5. Ankle swelling, the earliest visible sign of arthritis, was scored as 1. Thereafter, footpad swelling occurred and was scored as 2. Subsequent swelling of 1 or more digits resulted in a score of 3. The thickness of each hind paw was measured using a thickness gauge (Mitutoyo) and expressed in mm. Clinical signs of arthritis and body weight were monitored prior to immunization and then throughout the disease process.

### Behavior indicating pain

Mechanical and thermal hypersensitivity in the hind paws was assessed as an indicator of secondary hyperalgesia remote from the inflamed ankle joint. Changes in hind paw mechanical withdrawal thresholds were assessed by applying a series of calibrated von Frey filaments (0.4–15 gm; North Coast Medical) to the plantar surface of the hind paw according to the “up‐down” method 15. On each day of testing, animals were habituated for 15 minutes in individual transparent Plexiglas boxes with a wire mesh bottom in a temperature‐controlled room (22°C). Calibrated von Frey filaments were applied to the plantar surface of the hind paw for 3–5 seconds or until the paw was withdrawn. Mechanical thresholds of the left and right paws were assessed alternately. Each test started with application of the 2‐gm filament. Once a withdrawal response to a von Frey hair was established, the paw was retested starting with the filament below the one that elicited a withdrawal and subsequently with the remaining filaments in descending force sequence until no withdrawal occurred and then in ascending force sequence until a response was observed. This up‐down method was continued until the “k” value could be calculated (between 4 and 9 applications of the von Frey hairs). From this value, 50% withdrawal thresholds were calculated.

Heat hyperalgesia was assessed with a Plantar test apparatus (Ugo Basile) as previously described 16. Rats were habituated for 15 minutes to individual Plexiglas chambers placed on a glass floor in a temperature‐controlled room (22°C). A beam of radiant heat was applied onto the plantar surface of each hind paw, and paw withdrawal latencies were recorded. Withdrawal latencies were monitored 3 times in each paw alternately, and the latencies of both paws were averaged on each measurement day. At least 1 minute was allowed between consecutive measurements in the same paw, and a cutoff latency time of 20 seconds was used to avoid tissue damage. Baseline measurements were obtained prior to collagen immunization, and withdrawal latencies were then recorded on several days throughout the disease process.

### Intrathecal drug treatment

Two weeks prior to collagen immunization, intrathecal cannulae were implanted at the lumbar level in the spinal cord. While animals were under anesthesia, a small laminectomy was performed at the sixth thoracic vertebra and a flexible cannula was inserted under the dura mater and glued in place, such that the tip rested at the lumbar enlargement. The opposite end of the cannula was placed subcutaneously, and 11 days after immunization an osmotic minipump (Alzet model 2001) was connected to the cannula 5. CGRP receptor antagonist CGRP^8–37^ (1 mg/ml; Bachem) was delivered at 24 μl/day for 7 days (from day 11 to day 18 postimmunization), resulting in a dose of 24 μg/day as reported previously 17.

### Histology

Ankle joints were excised from rats with CIA and control rats that were perfuse‐fixed on days 7 and 18 postimmunization. The joints were postfixed for 1 week in the perfusion fixative, decalcified for 3 days in 10% formic acid with 0.5*M* trisodium citrate, and embedded in paraffin. Longitudinal sections (7 μm) were cut from the center of the ankle joint in the sagittal plane and stained with hematoxylin and eosin. Sections were examined under light microscopy for cellular infiltration, synovitis, and structural integrity.

### Retrograde neuronal labeling

To identify the cell bodies of ankle joint afferent neurons, 5 μl of 2% Fluoro‐Gold (FG; Fluorochrome) was injected intraarticularly using a Hamilton syringe into the ankle joint of the rats 7 days prior to the isolation of DRGs, as previously described 18. As intraarticular injection of FG does not produce bilateral labeling and collagen immunization affects both joints, we performed intraarticular injections of Fluoro‐Gold in both ankle joints.

### Tissue processing

On days 4, 7, and 18 postimmunization, naive rats, rats with CIA, and control rats were anesthetized with pentobarbital and transcardially perfused with 0.9% saline solution followed by 4% paraformaldehyde with 1.5% picric acid in 0.1*M* phosphate buffer (pH 7.4). L4 and L5 DRGs and lumbar spinal cords were excised, postfixed for 4 hours in the perfusion fixative (4°C), cryoprotected in 20% sucrose in phosphate buffer (0.1*M*, 4°C) for 48 hours, and frozen in OCT embedding compound (VWR International). DRG (15 μm) and spinal cord (20 μm) sections were cryostat‐cut and thaw‐mounted onto Superfrost Plus microscope slides (VWR International). In preliminary experiments all lumbar DRGs (L1–L6) were excised from naive rats and processed in the same way in order to elucidate the segmental distribution of DRG neurons innervating the ankle joint.

### Immunohistochemistry

Slides containing every sixth section of L4 and L5 DRGs from rats previously injected or not injected with FG were blocked with 1% normal goat serum (Jackson ImmunoResearch) for 1 hour and then incubated overnight with solutions of primary antibodies (sheep anti‐CGRP [1:800; Enzo], monoclonal rabbit anti–phospho‐p44/42 MAPK [ERK‐1/2] [p‐ERK] [1:400; Cell Signaling Technology], and/or Anti‐βIII Tubulin monoclonal antibody [1:1,000; Promega]) followed by incubation for 2 hours with solutions of appropriate secondary antibodies (Alexa Fluor 350 or Alexa Fluor 488 conjugated [Molecular Probes] or Cy3 conjugated [Jackson ImmunoResearch]). To ensure that the intraarticular injection of FG was correctly located, some FG‐labeled sections from each injected rat were incubated with fluorescein isothiocyanate–conjugated isolectin B4 (10 μg/ml; Sigma) for 1 hour, as it has been described that isolectin B4 staining is absent or expressed at very low levels in DRG neurons innervating joints 18, 19, 20. All antibodies were prepared in phosphate buffered saline (PBS) with 0.5% normal goat serum and 0.1% Triton X‐100 (Sigma).

Slides containing every sixth section of lumbar (L4 and L5) spinal cord were incubated overnight with a solution of the primary antibody rabbit anti–ionized calcium–binding adapter molecule 1 (anti–IBA‐1) (1:1,000; Wako) followed by incubation for 2 hours with a solution of the appropriate secondary antibody (Alexa Fluor 488 conjugated). All antibodies were prepared in PBS with 0.1% Triton X‐100.

In control experiments, primary antibody was omitted from some sections of spinal cords and DRGs; this resulted in complete abolition of staining. Slides were coverslipped with Vectashield mounting medium (Vector), and images were captured using a Zeiss Axioplan 2 fluorescence microscope.

### Immunofluorescence analysis

FG‐labeled DRG neurons were easily identifiable and considered to be FG labeled when cell profiles were clearly distinguishable from the faint, homogeneous staining of unlabeled neurons. To study the entire neuronal population in DRGs, sections not labeled with FG were used and neurons were identified by the specific neuronal marker βIII Tubulin. The number of FG‐labeled or βIII Tubulin–labeled neurons immunoreactive for CGRP and p‐ERK was counted (8–12 sections per rat and 4 sections per rat, respectively) and expressed as the percentage of total FG‐labeled or βIII Tubulin–labeled neurons. In spinal cord sections, IBA‐1+ profiles (as indicative of the number of microglial cells) were counted within fixed areas (6 in total) of 20 × 10^4^ μm^2^ on the lateral, central, and medial dorsal horns (3 sections per rat) 5, 21. Bilateral DRGs and dorsal horns were included in the analysis, and data were averaged for each rat.

### Release of CGRP from dorsal horn slices

On day 7 or day 18 postimmunization, horizontal lumbar dorsal horn slices (400 μm thick) with dorsal roots attached were obtained from the lumbar spinal cord of rats as previously described 22. One slice was obtained from each rat, mounted in the central compartment of a 3‐compartment chamber, and continuously superfused (1 ml/minute) with oxygenated Krebs solution (118 moles/liter NaCl, 4 moles/liter KCl, 1.2 moles/liter MgSO_4_, 1.2 moles/liter KH_2_PO_4_, 25 moles/liter NaHCO_3_, 2.5 moles/liter CaCl_2_, and 11 moles/liter glucose) containing 0.1% bovine serum albumin and 20 μg/ml bacitracin to minimize peptide degradation. The dorsal roots were placed in the lateral compartments and immersed in mineral oil to avoid dehydration. Before, during, and after electrical stimulation, 8‐ml fractions of superfusates were collected at 8‐minute intervals from the central compartment in glass tubes containing 0.1*M* acetic acid (VWR International). The release of CGRP was evoked by electrical stimulation of the dorsal roots for 8 minutes under conditions mimicking the transmission of noxious stimuli (C fiber strength; 20V, 0.5 msec, 10 Hz). Three 8‐ml fractions were collected before stimulation, one 8‐ml fraction was collected during electrical stimulation. and four 8‐ml recovery fractions were collected after stimulation.

To quantify CGRP content, superfusate samples were partially purified and desalted using Sep‐pak C18 reverse‐phase silica gel cartridges (Waters) previously conditioned with acetonitrile (VWR International) and 0.1% trifluoroacetic acid (TFA; VWR International). The peptide was then eluted from columns using a solution of acetonitrile/TFA (80:20). Eluates were dried by evaporation under nitrogen, reconstituted in 300 μl of sample buffer, and assayed for CGRP content by enzyme‐linked immunosorbent assay (standards 3.9–500 pg; SPI Bio). Data are expressed as the percentage of CGRP content in basal fractions.

### Statistical analysis

Differences between values in behavioral tests were analyzed with two‐way repeated‐measures analysis of variance (ANOVA) followed by Tukey's test. Immunohistochemistry and release data were analyzed by two‐way ANOVA followed by Tukey's test or by Student's *t*‐test as appropriate. Data are reported as the mean ± SEM. Differences between means were considered statistically significant when *P* values were less than 0.05.

## RESULTS

**Figure 1:**
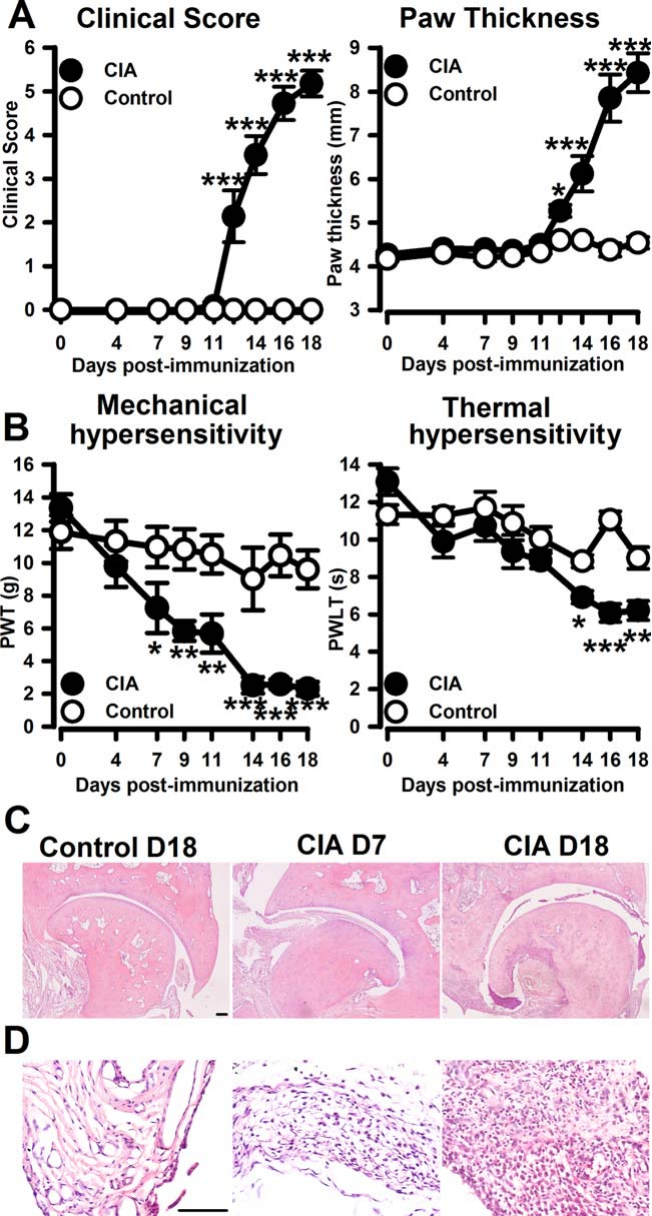
Collagen‐induced arthritis (CIA) is associated with development of hind paw inflammation and behavior indicating pain. **A,** Clinical scores and hind paw thickness. **B,** Hind paw mechanical and thermal thresholds. **C** and **D,** Photomicrographs of hematoxylin and eosin–stained ankle joints (**C**) and synovium (**D**). Values are the mean ± SEM (8–12 rats per group). * = *P* < 0.05; ** = *P* < 0.01; *** = *P* < 0.001 versus controls, by two‐way repeated‐measures analysis of variance followed by Tukey's test. Bars = 100 μm. PWT = paw withdrawal threshold; PWLT = paw withdrawal latency time.

While both clinical scores and paw swelling were apparent beginning 12–14 days after collagen immunization (Figure 1A), hind paw mechanical thresholds decreased significantly on day 7 postimmunization, decreasing further concomitant with establishment of clinical signs (days 14–18) (Figure 1B). Hind paw thermal thresholds decreased alongside the appearance of clinical signs (days 14–18) (Figure 1B). Histologic examination of the ankle joint revealed infiltration of inflammatory cells on day 7, which was exacerbated by day 18 (Figures 1C and D).

**Figure 2:**
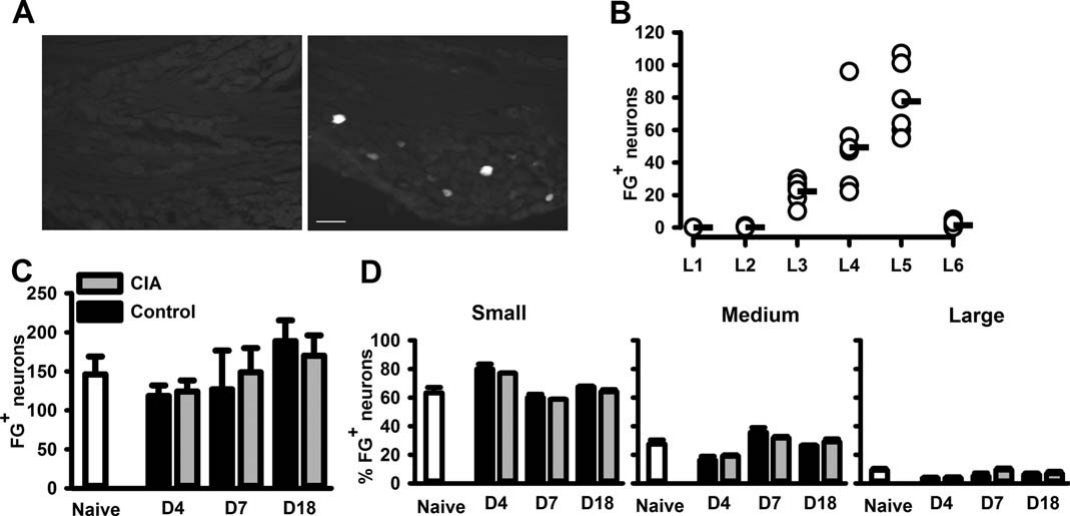
Development of collagen‐induced arthritis (CIA) induces no changes in numbers of ankle joint dorsal root ganglion (DRG) neurons retrograde‐labeled with Fluoro‐Gold (FG+). **A,** FG+ neurons detected in ipsilateral (right) but not contralateral (left) L5 naive rat DRGs. Bar = 100 μm. **B,** Distribution of FG+ neurons in naive rat L1–L6 DRGs (n = 6 rats). Circles represent individual values for each rat; horizontal lines indicate the mean. **C** and **D,** Number (**C**) and cell size distribution (**D**) of FG+ neurons in L4 and L5 DRGs during CIA development (n = 6 rats per group). Small size = 0–600 μm^2^; medium size = 600–1,200 μm^2^; large size = >1,200 μm^2^. Values are the mean ± SEM.

We hypothesized that increasing recruitment of sensory afferent fibers, which would be activated at the site of inflammation in the ankle joints and the hind paws, might contribute to the escalation of mechanical and thermal hypersensitivity associated with CIA. The numbers of neuronal cell bodies from the ankle joint retrograde‐labeled with Fluoro‐Gold (FG+) were quantified in naive rats, control (CFA emulsion–injected) rats, and rats with CIA. In naive rats, ∼85% of retrograde‐labeled neuronal cell bodies from the ankle joint were in the L4 and L5 DRGs, and the remaining 15% were in L3 DRGs (Figures 2A and B). FG+ neurons represented a mean ± SEM of 2 ± 0.4% and 4 ± 0.3% of the total population of L4 and L5 neurons (n = 6 rats), respectively. In rats with CIA, the number of FG+ neurons in the L4 and L5 DRGs on days 4, 7, and 18 following collagen immunization was similar to the number in naive rats and control rats (Figure 2C). In DRGs from naive rats, 60% of FG+ neurons had small‐size cell bodies, while 29% had medium‐size cell bodies and 9% had large‐size cell bodies (Figure 2D). This cell size distribution was not altered by CIA (Figure 2D). Thus, the majority of sensory neurons innervating the ankle joint were derived from the L4 and L5 DRGs and represented a small group of DRG neurons that remained unaltered by the progression of CIA.

**Figure 3:**
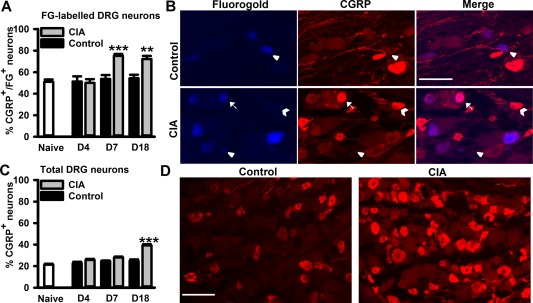
Development of CIA is associated with an increase in calcitonin gene‐related peptide (CGRP)–expressing neurons in lumbar DRGs. **A,** Quantification of CGRP+FG+ L4 and L5 DRG neurons during CIA development (n = 4–6 rats per group). **B,** CGRP+ neurons in L5 DRGs retrograde‐labeled with FG in control rats and rats with CIA at 18 days. **Arrows** indicate FG+CGRP+ neurons; **arrowheads** indicate FG+ neurons; **chevrons** indicate CGRP+ neurons. **C,** Quantification of CGRP+ neurons in whole L4 and L5 DRGs during CIA development (n = 4 rats per group). **D,** CGRP+ neurons in control rats and rats with CIA at 18 days. Values are the mean ± SEM. ** = *P* < 0.01; *** = *P* < 0.001 versus controls, by two‐way analysis of variance followed by Tukey's test. Bars = 100 μm. See Figure 2 for other definitions.

To investigate further the population of neurons innervating the ankle joint and the impact of CIA on them, expression of CGRP and p‐ERK, as markers for nociceptive neurons and neuronal activity, respectively, was quantified in FG‐labeled neurons and in the entire L4 and L5 DRG neuronal population (innervating sites outside the joint). At the earliest time point (day 4), there was no difference in the number of FG+ neurons that expressed CGRP in DRGs from naive or control rats or rats with CIA (Figure 3A). By day 7 following collagen immunization, there was a significant increase in the number of FG+CGRP+ DRG neurons compared to controls (Figure 3A). This increase in CGRP expression remained significant on day 18 following injection of collagen (Figures 3A and B). When the DRG neuronal population was considered as a whole (sensory neurons innervating both ankle and non‐ankle joints), the number of CGRP+ neurons was unaltered on days 4 and 7 following collagen immunization (Figure 3C), but was significantly increased on day 18 compared to controls (Figures 3C and D). Nonpeptidergic isolectin B4–positive neurons represented only 4.1 ± 0.9% of FG+ neurons (mean ± SEM) in naive DRGs (n = 4 rats) and remained unaltered in DRGs obtained 4, 7, and 18 days after collagen immunization (5.1 ± 1.0%, 7.1 ± 2.0%, and 6.5 ± 1.3%, respectively; n = 4 rats per group).

**Figure 4:**
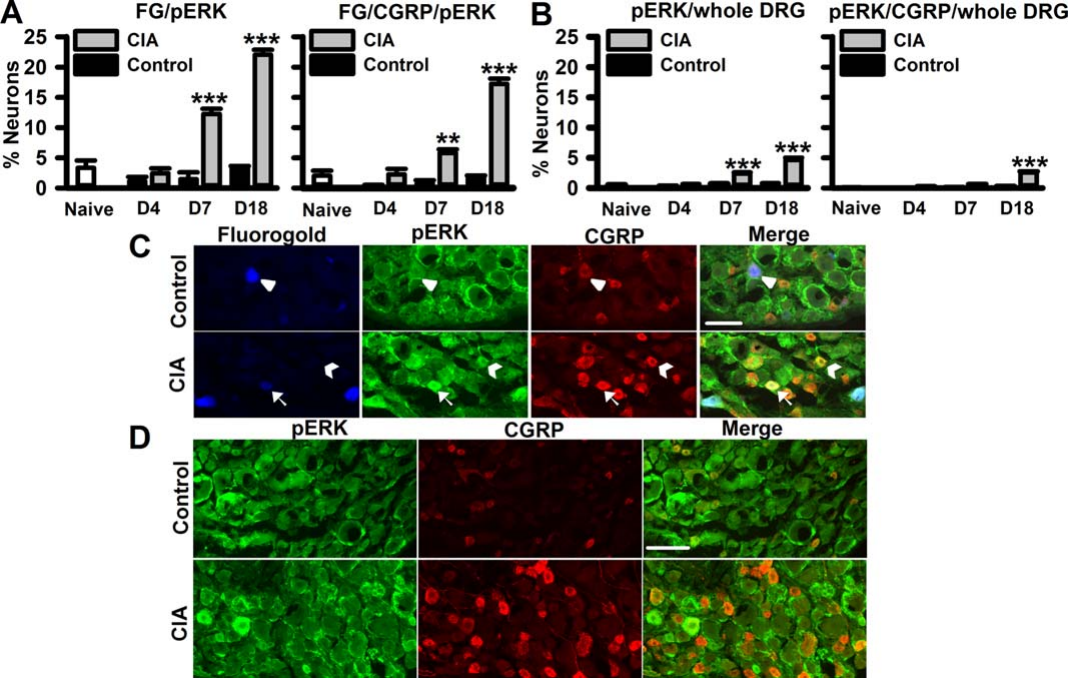
CIA development is associated with increased p‐ERK in lumbar DRGs. **A,** Quantification of FG+p‐ERK+ neurons and FG+ calcitonin gene‐related peptide–positive (CGRP+) p‐ERK+ neurons in L4 and L5 DRGs during CIA development. **B,** Quantification of p‐ERK+ neurons and p‐ERK+CGRP+ neurons in the whole neuronal population in L4 and L5 DRGs during CIA development. In **A** and **B,** n = 4–6 rats per group. **C,** Phospho‐ERK+ and CGRP+ neurons in L5 DRGs retrograde‐labeled with FG in control rats and rats with CIA at 18 days. **Arrows** indicate FG+p‐ERK+CGRP+ neurons; **arrowheads** indicate FG+CGRP+ neurons; **chevrons** indicate CGRP+p‐ERK+ neurons. **D,** Phospho‐ERK+ and CGRP+ neurons in the whole neuronal population in L4 and L5 DRGs in control rats and rats with CIA at 18 days. Values are the mean ± SEM. ** = *P* < 0.01; *** = *P* < 0.001 versus controls, by two‐way analysis of variance followed by Tukey's test. Bars = 100 μm. See Figure 2 for other definitions.

We also quantified the expression of p‐ERK in FG+ and FG+CGRP+ neurons. On day 4, there were no differences in p‐ERK expression in FG+ neurons among the 3 groups of rats (Figure 4A). By day 7, both FG+ and FG+CGRP+ neurons showed a significant increase in p‐ERK expression, which was further increased on day 18 following collagen immunization (Figures 4A and C). When the whole neuronal population in L4 and L5 DRGs was considered, there was a significant increase in p‐ERK expression in neurons on both day 7 and day 18 following collagen immunization (Figures 4B and D). However, p‐ERK expression in FG+CGRP+ neurons reached significance only on day 18 following collagen immunization compared to controls (Figure 4B).

These data indicate that beginning 7 days from collagen immunization, CGRP‐expressing sensory neurons start to be activated in the ankle joint. Activation of ankle joint afferents is then followed by activation of neurons that innervate tissues outside the joints with progression of CIA. As CGRP is a marker of nociceptors, these data provide a correlation between sensory neuron phenotype and increased nocifensive behavior on both day 7 and day 18 of CIA.

**Figure 5:**
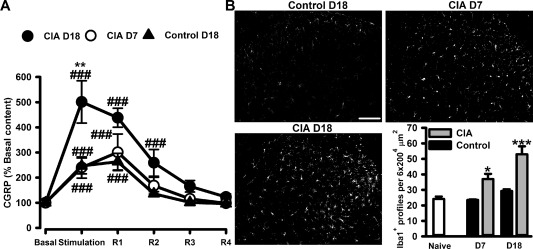
Enhanced release of calcitonin gene‐related peptide (CGRP) and significant microgliosis in the dorsal horn during collagen‐induced arthritis (CIA). **A,** Activity‐evoked release of CGRP from primary afferent fibers in the dorsal horn of control rats at 18 days and rats with CIA at 7 days and 18 days (n = 7 rats per group). Mean ± SEM basal peptide content was 7.8 ± 0.27 pg/8 ml (n = 9 rats). R1–R4 = recovery fractions. **B,** Left and top, Ionized calcium–binding adapter molecule 1 (IBA‐1)–positive profiles in control rats at 18 days and in rats with CIA at 7 days and 18 days. Bottom right, Quantification of IBA‐1+ profiles in the dorsal horn of control rats and rats with CIA during CIA development (n = 4–6 rats per group). Values are the mean ± SEM. * = *P* < 0.05; ** = *P* < 0.01; *** = *P* < 0.001 versus controls, by two‐way analysis of variance followed by Tukey's test. ### = *P* < 0.001 versus basal levels, by two‐way analysis of variance followed by Tukey's test. Bar = 100 μm.

In order to establish whether nociceptor activation in CIA resulted in increased input from the central terminals of these neurons in the spinal cord, we quantified the release of CGRP evoked by activity of primary afferent fibers. In the preparation of isolated dorsal horn with dorsal roots attached, obtained from control rats and from rats 7 and 18 days following collagen immunization, CGRP levels in dorsal horn superfusates were significantly increased following electrical stimulation of primary afferent fibers at noxious intensity, returning to basal levels after stimulation (Figure 5A). However, CGRP release in dorsal horns from rats with CIA at 18 days was significantly higher than that in dorsal horns from control rats at 18 days and from rats with CIA at 7 days (Figure 5A). Thus, despite the increase in retrograde‐labeled p‐ERK+CGRP+ neurons from the ankle of rats with CIA at 7 days, activity‐induced release of CGRP from their central terminals in the dorsal horn remained at control levels. Indeed, for enhanced activity‐evoked release of CGRP from the central terminals of nociceptors to occur, as was evident 18 days following induction of CIA, not only joint afferents but also neurons innervating tissues outside the joints must be recruited. In a further attempt to determine signs of enhanced central activity 7 days following collagen injection, we examined spinal microglia and observed significant microgliosis in dorsal horns from rats with CIA compared to those from control rats at 7 days (Figure 5B), with further enhancement in dorsal horns from rats with CIA at 18 days (Figure 5B).

**Figure 6:**
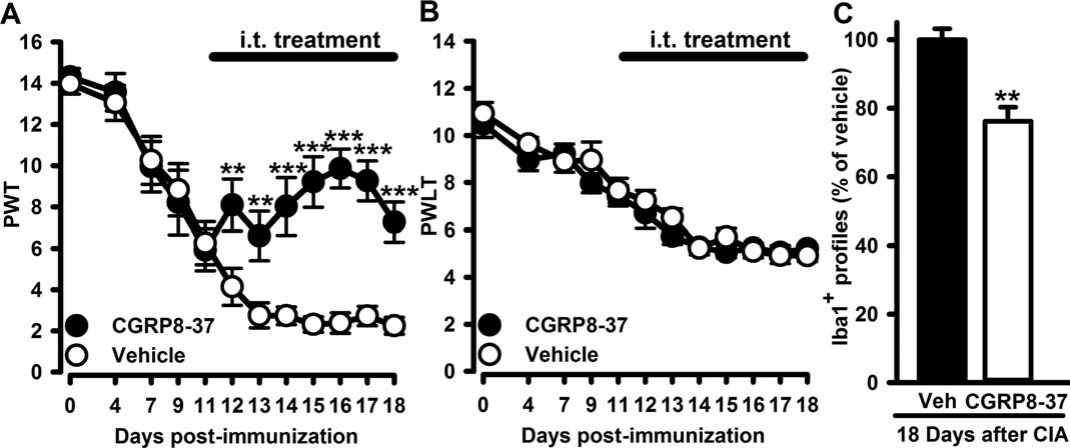
Intrathecal (IT) administration of a CGRP antagonist attenuates established mechanical hypersensitivity in CIA. **A** and **B,** Established mechanical hypersensitivity (**A**) but not heat hypersensitivity (**B**) attenuated by 7‐day intrathecal administration of CGRP^8–37^ (24 μg/day) (n = 9 rats per group). **C,** Quantification of IBA‐1+ profiles in the L4 and L5 dorsal horns at 18 days (n = 4 rats per group). Values are the mean ± SEM. ** = *P* < 0.01; *** = *P* < 0.001 versus vehicle (Veh)–treated controls, by two‐way repeated‐measures analysis of variance followed by Tukey's test (**A** and **B**) or by Student's *t‐*test (**C**). PWT = paw withdrawal threshold; PWLT = paw withdrawal latency time (see Figure 5 for other definitions).

Finally, in order to evaluate whether increased CGRP release from primary afferent fibers in the dorsal horn contributed to mechanical hypersensitivity and microgliosis, we intrathecally delivered a CGRP antagonist between days 11 and 18 of CIA. CGRP^8–37^ significantly attenuated mechanical hypersensitivity (Figure 6A) but not thermal hypersensitivity (Figure 6B) and produced a significant attenuation of microgliosis in the dorsal horn (Figure 6C).

## DISCUSSION

The main findings of this study are that before the appearance of overt clinical signs of CIA, mechanical hypersensitivity (allodynia) of the hind paw develops concomitant with inflammatory cell infiltration in the joint, activation of ankle joint nociceptors, and mild spinal microgliosis, but not paw swelling. However, with establishment of the disease, paw swelling and thermal hypersensitivity (hyperalgesia) accompany mechanical allodynia and, besides articular nociceptors, a significant number of primary afferent neurons innervating tissues outside the joint are activated. Furthermore, central changes in the dorsal horn of the spinal cord are readily detectable, including significant microgliosis and enhanced release of the pronociceptive peptide CGRP from nociceptor central terminals. Such release of CGRP is functionally relevant, as the delivery of a CGRP receptor antagonist to the lumbar spinal cord attenuates both mechanical allodynia and spinal microgliosis. Thus, similar to other forms of chronic pain 23, allodynia in CIA is likely to be maintained by a combination of central and peripheral mechanisms.

In the present study and in a previous study 5, we observed the development of allodynia before the onset of clinical signs of CIA in Lewis rats. Similarly, QB mice displayed allodynia before signs of arthritis in collagen antibody–induced arthritis 24. However, in DBA/1 mice, CIA allodynia developed along with the onset of clinical signs, although that study largely assessed pain in association with inflammation 4. These discrepancies highlight the importance of considering species and strain differences when selecting the most appropriate RA model for pain studies.

We observed that a small percentage of sensory neurons innervated the rat ankle joint, and their cell bodies were located in the L4 and L5 DRGs, consistent with previous findings in mice 25. These DRGs also contain neurons innervating additional sites including skin and muscle of the hind paw and the knee joint 18, 26, 27. The number of sensory neurons innervating the ankle joint was not altered by inflammation at any stage of CIA, but the expression of both the nociceptive peptide CGRP and the activation marker p‐ERK increased with the severity of disease, demonstrating that CIA is associated not only with the activation of these sensory neurons in the ankle joint, but also with the activation of neurons innervating areas outside this joint. For instance, the synovium of the ankle joint is highly innervated by CGRP‐expressing fibers, with enhanced expression of CGRP reported in both the joint and DRGs following adjuvant‐induced arthritis 28. Furthermore, as phosphorylation of ERK occurs in sensory neurons following peripheral noxious stimulation, the activation of this intracellular pathway is indicative of nociceptive neuron activation 29, 30. The increase in nociceptive sensory neuron activation, together with the development of allodynia and the exacerbation of the disease reported herein, provide a mechanism that links pain and inflammation. However, given that nociceptive sensory neuron activation and the development of allodynia occurred in the absence of paw swelling, our data also suggest that pain hypersensitivity in the early phase of CIA precedes macroscopic inflammation.

Joint afferents are highly responsive to mechanical stimuli 6; thus, sensitization of these fibers likely drives the central changes that underlie the early development of mechanical allodynia during CIA. Nevertheless, even though sensory neurons up‐regulated nociceptive peptide expression and displayed the presence of activation markers in the early development of allodynia, their activity was not sufficient to detect increased CGRP peptide release from their central terminals in the spinal dorsal horn. It is possible that the sensitivity of our assay does not allow for detection of small changes in CGRP content that might have occurred. In any case, alongside CGRP, the occurrence of glutamate and SP release from the central terminals of primary afferent fibers in the dorsal horn may contribute to behavioral allodynia 7. In order for primary afferent enhanced input to become detectable in our experimental system, the disease had to reach advanced phases with evident inflammation and swelling in the joints. Microglia appeared to be a more sensitive indicator of spinal cord changes in CIA than increased primary afferent input, as microgliosis was detected concomitant with the manifestation of allodynia and before the onset of clinical signs.

Our data suggest that pain development in CIA involves not only inflammation and changes in the joint, but also alterations in central pain processing that are dependent on pathologic inflammatory mechanisms. Thus, subtle collagen‐induced changes in the joint cartilage lead to an inflammatory milieu, including enhanced cytokine and prostaglandin release, which results in the sensitization of sensory neurons locally and mild microgliosis in the spinal cord during the early phase of CIA allodynia. Spinal microgliosis gradually increases with escalating activation of nociceptive neurons by inflammatory mediators in the joint with CIA. These mechanistic data are particularly relevant in the context of CIA as a model of RA, in view of the suggestion that activation of central interleukin‐1 signaling contributes to fatigue in RA patients in whom the most likely source of this cytokine are the microglia 31. Peripheral and central changes grow in both incidence and significance in the later phases of CIA allodynia and hyperalgesia, which are characterized by an evident increase of noxious input from the periphery to the spinal cord. In CIA, central changes such as microgliosis and increased afferent input play a critical role in mediating allodynia as demonstrated by the analgesic effect of a centrally delivered CGRP receptor antagonist. Nevertheless, the small though significant microglial inhibition through putative microglial CGRP receptors 32, 33 is not likely to be the primary mechanism of this antagonist, and attenuation of microgliosis is probably a reflection of neuronal receptor blockade 34, 35.

While we observed no changes in paw swelling following CGRP antagonist treatment (data not shown), spinal mechanisms can regulate the extent of peripheral inflammation in arthritis models. For example, spinal manipulation of microglial signaling by inhibiting p38 MAPK reduces joint inflammation and disease progression 36, with levels of spinal cytokines such as tumor necrosis factor α (TNFα) regulating neuronal responses to mechanical stimulation of the inflamed joint 37. It is relevant that neutralization of TNFα in RA patients inhibits pain before reducing inflammation in the joint 38, possibly through inhibition of central sensitization. In our study, the mechanisms underlying CIA thermal hyperalgesia, which develops concomitant with the onset of swelling and is unaltered by a centrally delivered CGRP antagonist, remain to be established. These mechanisms may reside peripherally and may arise from activation of different subsets of peripheral and spinal neurons 39. Indeed, while changes in mechanical sensitivity are a major feature of central sensitization, peripheral sensitization plays a major role in the alteration of heat sensation 13.

In conclusion, while RA results from immune‐mediated inflammation and damage to the joint, pain in RA may not simply be the result of joint inflammation. Indeed, pain may be experienced by patients before clinical signs permit confirmation of the diagnosis 1. We suggest that pain therapy in RA can be improved by developing treatment strategies that take into account the association of NSAIDs with molecules that stop central changes, for example, inhibitors of microglial cell reactivity and CGRP receptor antagonists or antibodies. Most of the currently available CGRP antagonists, which were developed for migraine, do not readily enter the central nervous system (CNS) 40. However, whether CNS penetration is critical for their antimigraine effect is still under debate 40. Our study suggests that CNS‐penetrating CGRP antagonists, when available, may be effective analgesics in RA.

## AUTHOR CONTRIBUTIONS

All authors were involved in drafting the article or revising it critically for important intellectual content, and all authors approved the final version to be published. Dr. Malcangio had full access to all of the data in the study and takes responsibility for the integrity of the data and the accuracy of the data analysis.

**Study conception and design**. Chapman, Malcangio.

**Acquisition of data.** Nieto, Clark, Grist.

**Analysis and interpretation of data**. Nieto, Clark, Malcangio.
